# Density-based clustering of static and dynamic functional MRI connectivity features obtained from subjects with cognitive impairment

**DOI:** 10.1186/s40708-020-00120-2

**Published:** 2020-11-26

**Authors:** D. Rangaprakash, Toluwanimi Odemuyiwa, D. Narayana Dutt, Gopikrishna Deshpande, Alzheimer’s Disease Neuroimaging Initiative

**Affiliations:** 1 Athinoula A. Martinos Center for Biomedical Imaging, Massachusetts General Hospital, Charlestown, MA USA; 2 Department of Radiology, Harvard Medical School, Boston, MA USA; 3 Division of Health Sciences and Technology, Harvard University and Massachusetts Institute of Technology, Cambridge, MA USA; 4 Division of Engineering Science, Faculty of Applied Science & Engineering, University of Toronto, Toronto, ON Canada; 5 Department of Electrical Communication Engineering, Indian Institute of Science, Bangalore, India; 6 AU MRI Research Center, Department of Electrical and Computer Engineering, Auburn University, 560 Devall Dr, Suite 266D, Auburn, AL 36849 USA; 7 Department of Psychological Sciences, Auburn University, Auburn, AL USA; 8 Alabama Advanced Imaging Consortium, University of Alabama Birmingham, Alabama, USA; 9 Center for Health Ecology and Equity Research, Auburn University, Auburn, AL USA; 10 Center for Neuroscience, Auburn University, Auburn, AL USA; 11 School of Psychology, Capital Normal University, Beijing, China; 12 Key Laboratory for Learning and Cognition, Capital Normal University, Beijing, China; 13 Department of Psychiatry, National Institute of Mental Health and Neurosciences, Bangalore, India

**Keywords:** Functional MRI, Brain networks and dynamic connectivity, Cognitive impairment and alzheimer’s disease, Unsupervised learning and clustering, DBSCAN, OPTICS

## Abstract

Various machine-learning classification techniques have been employed previously to classify brain states in healthy and disease populations using functional magnetic resonance imaging (fMRI). These methods generally use supervised classifiers that are sensitive to outliers and require labeling of training data to generate a predictive model. Density-based clustering, which overcomes these issues, is a popular unsupervised learning approach whose utility for high-dimensional neuroimaging data has not been previously evaluated. Its advantages include insensitivity to outliers and ability to work with unlabeled data. Unlike the popular *k*-means clustering, the number of clusters need not be specified. In this study, we compare the performance of two popular density-based clustering methods, DBSCAN and OPTICS, in accurately identifying individuals with three stages of cognitive impairment, including Alzheimer’s disease. We used static and dynamic functional connectivity features for clustering, which captures the strength and temporal variation of brain connectivity respectively. To assess the robustness of clustering to noise/outliers, we propose a novel method called recursive-clustering using additive-noise (R-CLAN). Results demonstrated that both clustering algorithms were effective, although OPTICS with dynamic connectivity features outperformed in terms of cluster purity (95.46%) and robustness to noise/outliers. This study demonstrates that density-based clustering can accurately and robustly identify diagnostic classes in an unsupervised way using brain connectivity.

## Introduction

Since the successful emergence of functional neuroimaging, a new barrier has surfaced: can a strong correlation be established between brain activity (as measured by functional magnetic resonance imaging [fMRI]) and the cognitive state of an individual? More specifically, can we accurately classify neurological diseases based on fMRI data? In response to this, machine learning classifiers have been employed on neuroimaging features to generate models that, within some accuracy, predict the cognitive and disease states to which new data belong [1–11].

There are two major categories of machine learning classification techniques: supervised and unsupervised. Supervised learning, commonly used in fMRI studies, involves splitting the dataset into training and test data. Each member of the training data is given a ‘label’ as to which class (or group) it belongs to. The classifier is then ‘trained’ on this data to determine a generalized model (thus ‘supervised’ learning). Then, the classification accuracy is measured by testing the model on the test data with known labels. Conversely, in unsupervised learning, patterns within the entire dataset are used to ‘cluster’ the data without any pre-assigned labels, and cluster purity is measured against the known ground-truth, post hoc, instead of an accuracy. As such, unsupervised learning is agnostic to pre-assigned labels, and thus determines inherent classes instead of fitting a model based on classes provided by us. Although the ‘cluster purity’ given by clustering is, in principle, the same as the ‘classification accuracy’ given by supervised classifiers (both give the percentage of correct classifications as against the known ground truth), we would use the term ‘cluster purity’ in this work, so as to highlight that this metric was obtained through unsupervised clustering, and not through conventional supervised classification.

FMRI studies generally use supervised learning methods to classify disease or cognitive states [1–3, 12, 13]. Unsupervised learning methods are generally used for spatially and temporally clustering voxel signals to localize brain activity [10, 11, 14–20]; for dimensionality reduction or feature selection before applying a supervised learning algorithm to the data [13, 21–24]; or for mapping specific brain patterns to a cognitive state [6, 25]. A few reports have adopted unsupervised learning methods as state classifiers across individuals. For example, one study used the one-class support vector machine (SVM) to determine the boundary around healthy control subjects and to classify outliers based on their distance from this boundary. However, such methods are highly susceptible to noise and do not work well with the high-dimensional data common in fMRI [26].

Research on clustering of subjects using unsupervised learning methods is still limited in fMRI literature, to the best of our knowledge. Diagnostic labels are often predetermined in neuroimaging studies, and most studies often stick to this labeling, because of which supervised classifiers are more popular. However, the main advantage of unsupervised learning is that it requires no a priori knowledge of categorical labels, thus reducing the problem of selection bias described by Demerci et al. [5]. This allows for the learning of more complex models, pattern discrimination and the identification of hidden states [27] among others. Unsupervised learning also does not have the issue of overfitting the data, which largely plagues supervised learning models. The overfitting nature of supervised learning models results in high performances on the data being used, but much lower performance when tested on an independent dataset. Unsupervised learning does not encounter this issue from the very nature of its formulation since learning is done in an entirely blind manner. With unsupervised learning, what you see is what you get. In this work, we demonstrate the utility and feasibility of the unsupervised learning approach of density-based clustering, applied on resting-state fMRI (rs-fMRI) data to cluster disease states in a noise-robust manner.

Clustering is an important technique of grouping objects that are similar to each other and isolating them from those that are dissimilar. Clusters are formed with features having minimum intra-cluster distance and maximum inter-cluster distance [28]. Clustering has found wide application in many fields including, but not limited to, the recovery of information, recognition of natural patterns, the analysis of digital images, bioinformatics, data mining, taxonomy, DNA microarray analysis, and many others [29–34]. The performance of most clustering algorithms, however, is highly sensitive to their input parameters, such as the number of clusters in k-means clustering [35]. For effective performance, one almost needs a priori knowledge of these parameters. Although there are a variety of clustering algorithms, density-based clustering is one of the most computationally effective methods in clustering large-scale databases [36]. Unlike *k*-means clustering, density-based clustering techniques do not need the user to specify the number of clusters since the algorithm determines that by itself. Using a local proximity measure, clusters are automatically formed by points in higher density regions, well separated by lower density regions. In this work, we focus on two of the most popular density-based clustering methods: Density-Based Spatial Clustering of Applications with Noise (DBSCAN) and Ordering Points to Identify the Clustering Structure (OPTICS). They require few input parameters, need no a priori user choices, detect outliers, and effectively detect clusters of arbitrary shapes [37, 38].

DBSCAN is a single-scan technique that makes no presumptions regarding the distribution of data. In addition to clustering the data, the algorithm also detects outliers. The OPTICS algorithm was developed by Ankerst et al. [38] to address DBSCAN’s major shortcoming that it cannot detect clusters with different local densities. OPTICS is a non-rational, data-independent representation of the cluster structure that displays important information regarding the distribution of the data. It is capable of identifying all possible clusters of varying shapes. Advantages of OPTICS over DBSCAN are that it eliminates free parameter choices needed in DBSCAN, is relatively insensitive to noise and can detect clusters of different densities [38].

In fMRI data, noise is any signal variation that is not contributed by neuronal activity. It may arise from head movements during scanning, scanner limitations, thermal noise, or other sources [39, 40]. The signal-to-noise ratio (SNR) is a measure of how much signal is present in the data relative to noise. Higher SNR values indicate higher quality data with low amounts of noise. However, much progress has been made in detecting and minimizing noise artifacts from fMRI data, like high-pass filtering, motion correction and other pre-processing steps [39, 41]. Some noise is always prevalent in the data. It is important that learning methods perform well even when given lower quality data. In this work, we assess the robustness of clustering to such noisy variations.

Resting-state fMRI (rs-fMRI) involves the collection of fMRI data from subjects while at rest in the scanner, with eyes open and mind left to wander. It is characterized by correlations across various regions of the brain, also called functional connectivity (FC). FC estimated from rs-fMRI is extensively used to study brain networks, and prior works have identified alterations in FC in subjects with various psychiatric and neurological conditions (please see [42–45] for a review). We have used functional connectivity features in this work.

Static Functional Connectivity (SFC) refers to the strength of connectivity between two brain regions and is quantified using Pearson’s correlation coefficient between pairs of fMRI time series. It evaluates the temporal correlation between fMRI time series of two brain regions, giving one correlation value over the entire duration of the scan. On the other hand, variance of Dynamic Functional Connectivity (DFC) [46–48] captures time-varying connectivity and is obtained using sliding-window Pearson’s correlation between pairs of brain regions. Although SFC is the most widely used measure of fMRI connectivity, DFC is emerging as an important measure having certain unique special properties [42]. SFC and DFC provide characteristically different information regarding the relationship between two brain regions. While SFC gives the strength of connectivity or co-activation, DFC gives the variation of connectivity over time (please refer to Hutchison et al. [42] for a review of dynamic connectivity). In this work, in addition to evaluating the performance of DBSCAN and OPTICS clustering approaches, we compared the performance of SFC and DFC features. We explore which of these two popular measures is more suitable for clustering in cognitive impairment.

In this work, SFC and DFC features were obtained from 132 subjects with progressive stages of cognitive impairment (early mild cognitive impairment [MCI], late MCI and Alzheimer’s disease), along with matched healthy controls. Cognitive impairment [49] is a spectrum disorder ranging from mild to severe symptoms. There has been enormous interest in identifying subgroups of cognitive impairment representing relatively homogeneous symptoms [49]. In this work, we performed clustering and assessed how well the clustered subject groups matched with clinically diagnosed groups. If this approach yields impressive and reliable results, it would advance clinical diagnosis and classification of cognitive impairment.

In this work, we assessed the performance of DBSCAN and OPTICS, in combination with SFC and DFC features, in accurately clustering three groups of cognitive impairment (including Alzheimer’s disease) and a matched healthy control group. Higher clustering performance need not necessarily imply higher robustness to noisy variations in the data. It is possible that a high cluster purity is obtained, but that high performance is not sustained if noise in the data increases. Noise robustness is very important for two reasons: (i) biomedical data, especially brain imaging data, are vulnerable to a large number of unknown sources of variability and known sources of noise, and (ii) the data used in any study is only a representative sample of the general population, and hence generalizability and inter-subject variability are imminent issues; therefore, if the results have to be applicable in a real-world setting, then it has to be robust to such variability. Thus, to assess robustness, we developed a novel technique called ‘recursive clustering using additive noise’ (R-CLAN). Additionally, since well-formed clusters are partly defined by how well the clusters are separated in the feature space, we propose the ‘separation index’ to supplement the R-CLAN outcome, as a measure of OPTICS’ performance.

The organization of the paper is as follows: section-2 describes the methods used for grouping the dataset into clusters and our methods to assess the algorithms’ robustness; section-3 presents the results with clustering performances and their robustness; section-4 evaluates and discusses the findings, and highlights possible future work; and section-5 provides concluding remarks.

## Methods

### Data acquisition and pre-processing

The fMRI data used in this work were obtained from the Alzheimer's Disease Neuroimaging Initiative (ADNI) database (https://www.adni-info.org/). ADNI is a multisite, longitudinal study employing imaging, clinical, bio-specimen and genetic biomarkers in healthy elders as well as in individuals with early mild cognitive impairment (EMCI), late MCI (LMCI) and Alzheimer’s disease (AD). The ADNI was launched as a public–private partnership, led by Principal Investigator Michael W. Weiner, MD. The primary goal of ADNI has been to test whether serial neuroimaging and other biological markers can be combined with clinical and neuropsychological assessment to measure the progression of MCI and AD. For up-to-date detailed information, please see www.adni-info.org. Table 1 provides the demographics of the subjects used by us.

**Table 1:** Basic demographics

Variable	Control	Early MCI	Late MCI	AD
Age, years				
Mean	74.5	72.2	71.4	73.1
Median	73.8	72.9	72.3	74.5
SD	5.9	5.9	8.6	7.4
Range	20.5	26.8	30.9	30.6
Gender, no. of subjects				
Male	15	18	21	13
Female	21	16	13	16

A total of 132 subjects were considered from phase-1 of the database: 35 control subjects, 34 EMCI, 34 LMCI and 29 AD patients. RS-fMRI data were acquired in 3 T Philips MR scanners using a *T*2* weighted single shot echo-planar imaging (EPI) sequence with 48 slices and the following parameters: repetition time (TR) = 3000 ms, echo time = 30 ms, flip angle = 80°, voxel size = 3.3125 × 3.3125 × 3.3125 mm^3^, and 140 temporal volumes in each run. Field of view parameters were the following: Right–Left (RL) = 212 mm, Anterior–Posterior (AP) = 198.75 mm, and Foot-Head (FH) = 159 mm. Anatomical images were acquired using magnetization-prepared rapid acquisition gradient echo (MPRAGE) sequence for overlay and localization (TR = 6.8 ms, echo time = 3.1 ms, voxel size: 1.11 × 1.11 × 1.2 mm^3^, flip angle = 9°, field of view: RL = 204 mm, AP = 253 mm, FH = 270 mm).

Prior to pre-processing, first five volumes of the fMRI time series were discarded to allow for MR scanner equilibration. Standard rs-fMRI data preprocessing steps were performed on the raw data (realignment, normalization to MNI space, detrending, regressing out nuisance covariates [6 head motion parameters, white-matter signal and cerebrospinal fluid signal] using SPM8 [50] and DPARSF [51] toolboxes in the Matlab^®^ environment. Mean fMRI time series were then extracted from 200 functionally homogeneous regions-of-interest (ROIs) obtained through spectral clustering (Craddock-200 atlas, [52]).

### Obtaining connectivity features

SFC was obtained between all pair-wise ROIs using Pearson’s correlation coefficient, giving a 200 × 200 SFC matrix per subject. Multivariate N-way analysis of covariance (MANCOVAN) was used to perform statistical tests between SFC features of all groups, controlling for age, gender and head motion. The top-100 (i.e. lowest *p* values) significant features were selected for further analysis, that is, a 132 × 100 (subjects × features) SFC matrix was used for clustering. We chose a fixed number of features, because we did not want the difference in number of features (between SFC and DFC) to impact clustering performance. DFC was obtained using sliding-windowed Pearson’s correlation [46–48], giving a time series of correlation values. The variance of these values over time is a measure of how much the connectivity varies over the duration of the scan, which was the measure we used further. The width of the sliding windows was not chosen arbitrarily, like in most studies, but was instead evaluated objectively wherein the window lengths were determined adaptively using the augmented Dickey-Fuller (ADF) test [46–48], an analytical test based on timeseries stationarity. Overlapping windows were used, with successive windows differing by one time point. Similar to SFC, a 132 × 100 variance of DFC matrix was obtained, which was then used in clustering. Please refer to Additional file 1: Tables S1 through S3 for the list of all the top-100 significant SFC and DFC connectivity paths from which the features were extracted for further analysis.

### The DBSCAN algorithm

For implementation of DBSCAN and OPTICS, we wrote custom Matlab scripts using Daszykowski’s functions (https://www.chemometria.us.edu.pl/index.php?goto=downloads). Here, we give a brief overview of the DBSCAN algorithm, as described by Ester et al. [37]. The idea, in its simplest form, is that the density of points within a cluster must exceed a certain global threshold. Regions of density below this threshold are considered as noise or outliers. Two input parameters, *ε*, the minimum radius of a cluster, and *MinPts*, the minimum number of points required in a cluster, form the global threshold measures. In distinguishing regions of data by density, the following terms are used:

- *ε-neighborhood*: For a point *p* in the cluster, its *ε*-neighborhood is the set of points contained within the radius *ε* from that point.
- *Core point*: A point is a core point if its *ε*-neighborhood contains at least *MinPts* points.
- *Border point*: A point is a border point if there are less than *MinPts* number of points in its *ε*-neighborhood.
- *Density-reachable*: A point *q* is directly density-reachable from point *p* if *p* is a core point, and *q* is within the *ε*-neighborhood of *p*. In general, *q* is density-reachable from *p* if there is a chain of points from *p* to *q* such that each successive point is directly density-reachable from the previous point.
- *Density-connected*: Two points, *p* and *q*, are density-connected if there exists a third point from which both are density-reachable.

Starting at a core point *p*, DBSCAN gathers all the density-reachable points of *p* into a single cluster *C*. The procedure is then repeated again with all the new core points in *C* until the cluster is completely surrounded by border points from which no other point is density-reachable. DBSCAN then repeats the process again for any unclustered points in the database until all points have been processed. Finally, the points within a cluster will all be density-connected with each other, and the points lying completely outside these clusters will be considered as outliers.

### Determination of radius in DBSCAN algorithm

As with the other clustering algorithms, DBSCAN is sensitive to its input parameters, particularly to *ε*. As noted in Tench et al. [53], if *ε* is too large, the algorithm will not be as discriminative and may include outliers in its clusters, and if too small, clusters may not be detected at all. Consequently, Ester et al. [37] proposed the method of *k*-distance graphs to determine *ε*. In this method, all the data points are ordered according to their distance d from their *kth* nearest neighbor (setting *k* = *MinPts*), and a *k*-distance graph is formed by plotting *d* against the ordered points in the feature space. By visual inspection, *ε* is chosen to be the value *d* at which an ‘elbow’ occurs in the plot. However, this method does not provide dependable *ε* values [54], although it effectively identifies the range of values where one can search for a more optimal *ε* value.

We modified their approach by first obtaining an initial estimate of *ε* using the *k*-distance method, and then performing DBSCAN for all *ε* values ranging from zero to twice this initial estimate, resulting in different number of clusters for different *ε* values. Finally, we searched for the number of clusters that spanned the largest range of *ε* values, since this would identify the number of clusters that were most stable among all choices of the parameter *ε*. We assigned the mean *ε* value within this range as the final *ε* value for performing DBSCAN clustering. The rationale behind this approach was that those number of clusters that spanned the largest *ε* range would represent the most stable and true separation between the clusters.

This process can be visualized through what we term as the epsilon plot (Fig. 3, shown later in the results section), which is a graph of the number of clusters identified by DBSCAN against the range of *ε* values. Since the number of clusters is a discrete set of values, the plot is characterized by a series of horizontal steps, where a step corresponds to the set of *ε* values that give rise to that number of clusters. As described earlier, the *ε* value corresponding to the midpoint of the widest step was chosen as the final *ε* value used in the DBSCAN clustering.

### The OPTICS algorithm

Here, we provide a brief overview of OPTICS as described by Ankerst et al. [38]. OPTICS is effectively an extension of the DBSCAN algorithm that uses a range of *ε* values, rather than a single, global threshold, to identify clusters of different local densities. Akin to DBSCAN, OPTICS uses input parameters *MinPts* and ‘generating distance’ *ε*; however, *ε* is now a maximum threshold value. We use *ε*′  to denote the range of radius values used by OPTICS, where 0 < *ε*′ < *ε*. To understand the algorithm, two terms are defined in addition to those used in DBSCAN:

- *Core distance*: The core distance of a point *p* is the distance *ε*′  between *p* and its *MinPts*’ neighbor such that *p* is a core point with respect to *ε*′ . If this *ε*′  value is greater than *ε*, then the core-distance is undefined.
- *Reachability distance (of p from s)*: If *s* is not a core point with respect to *ε*, the reachability distance is undefined. Otherwise, the reachability distance of a point *p* from a point *s* is the maximum of *s*’s core distance and smallest distance such that *p* is density-reachable from *s*.

OPTICS works on the principle that to accurately distinguish between different densities of clusters; higher density clusters must first be found before lower density clusters are identified. Hence, OPTICS orders data points based on increasing *ε*ˈ value, since smaller *ε* values indicate higher density regions. This ordered list is part of the output of OPTICS. As in DBSCAN, points not belonging to any cluster are considered outliers. Unlike DBSCAN, however, OPTICS does not explicitly assign cluster memberships; rather, points are ordered based on their reachability and core distance values. In addition, OPTICS is relatively insensitive to *ε* and *MinPts*. As long as a large enough value is chosen for *ε*, and a choice of around 10–20 is made for *MinPts* (according to [38]), consistent cluster structures will be identified. This makes implementing OPTICS practically non-arbitrary and choice-free.

### Separation index to evaluate robustness of reachability plot in OPTICS clustering

**Fig. 1:**
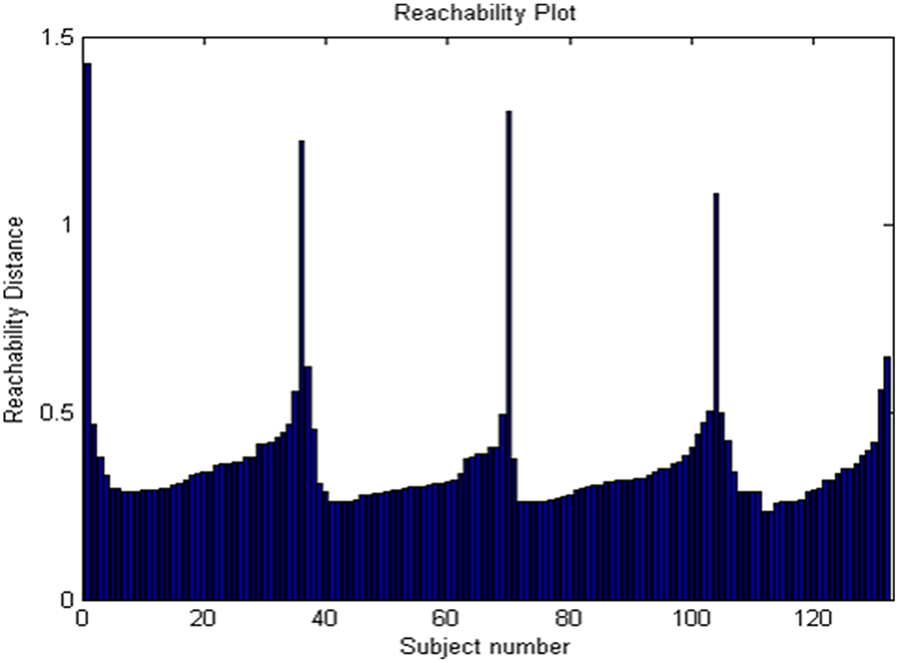
Example illustration of the reachability plot obtained in OPTICS

To assess the clustering structure in OPTICS, a reachability plot is popularly used [38]. The reachability distance is plotted for each data point, ordered according to the ordered-list output of OPTICS. Figure 1 gives an example illustration of the reachability plot defined by four clusters. A series of peaks and valleys characterize the plot, where a valley indicates a cluster. This plot effectively indicates the cluster membership of each point, although further steps are performed in OPTICS to formally define each cluster. Further details can be found in [38]. Since obtaining the reachability plot is an intermediate step in OPTICS clustering, the success of the method critically depends on the reachability plot. Consequently, assessing the robustness of the plot is indirectly an assessment of the outcome of OPTICS clustering.

To assess the performance of OPTICS on both SFC and DFC features using the reachability plot, we devised a novel measure called the OPTICS separation index. Leaving out the first and last points of the reachability plot, we defined the separation index as the ratio of the mean of peak heights to the mean value of points between the peaks in the valleys. For example, suppose there exists peaks at points *i* and *j* in the reachability plot, with cluster-*k* defined by the valley bounded by these peaks, then the separation index for cluster-*k* is defined as:

1 $${S}_{k}=\frac{\frac{1}{2}\left(RP\left(i\right)+RP\left(j\right)\right)}{\frac{1}{j-i-1}\sum_{m=i+1}^{j-1}RP\left(m\right)},$$

where *S*_*k*_ is the separation index for the *kth* group, and *RP(i)*, *RP(j)* are the values of the reachability plot. The final separation index is an average of the separation indices obtained for each cluster. In simple terms, the separation index evaluates the ratio of the peak heights to the average of the baseline heights. A higher separation index value indicates that the peaks are relatively much larger than the baseline; hence, obtaining clusters using a threshold on the reachability plot would then be less prone to noise from outliers. This is an indirect measure of the robustness of OPTICS clustering.

### Assessing robustness using additive noise

Clustering could be defined as robust if it can maintain its performance in the presence of higher noise levels compared to noiseless data. Thus, robustness is a measure of how well the clustering would be performed if lower quality data were given. A clustering method providing higher cluster purity does not necessarily imply that it is more robust to noisy variations in the data. It is possible for a clustering method to provide high performance yet be more sensitive to outliers and noise.

To assess the robustness of DBSCAN and OPTICS, each in association with SFC and DFC input features, we devised a novel approach called Recursive CLustering using Additive Noise (R-CLAN). Here, white Gaussian noise was successively added to SFC (or DFC) features such that the SNR value of the subsequent corrupted features decreased by 1 dB per iteration, with SNR values starting from 100 dB in the first iteration. In this context, the traditional definition of SNR was used, that is, SNR = 10 × log(*S/N*), where * S* was the signal power (mean squared value of all the original SFC [or DFC] values),* N* was the noise power (mean squared value of additive Gaussian noise), and the logarithm being taken to the base of 10. An SNR value of 100 dB indicates a high amount of signal relative to noise (higher quality data), whereas a value of 0 dB indicates an equal amount of noise and signal in the data, making it difficult to distinguish signal from noise (lower quality data).

**Fig. 2:**
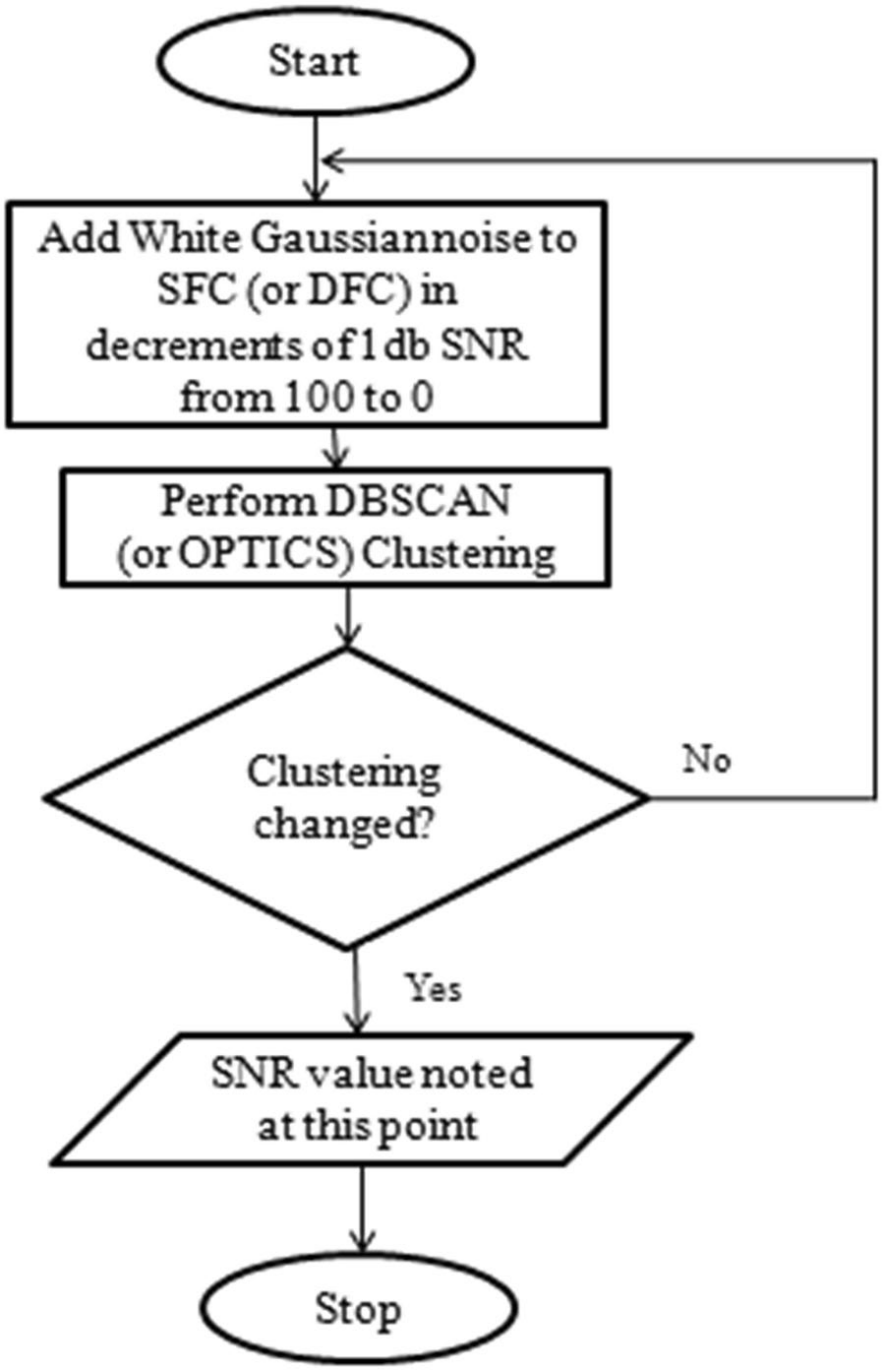
Flowchart for assessing robustness using additive noise

Starting with data having an SNR of 100 dB, we performed DBSCAN and OPTICS clustering at each SNR value, decreasing the SNR by 1 dB in each iteration and terminating only when the clustering structure had changed (i.e. the cluster purity value changed from the original at 100 dB). That means, we stopped at that SNR value when at least one subject in one of the groups was clustered into another group, thus changing the groups’ structure. At this point, the terminating SNR value was taken as a measure of robustness. The lower the SNR value, the more noise that was present in the data, thus more robust the algorithm would be in combination with the input features (SFC or DFC), since the low SNR indicates a higher tolerance for noise. Figure 2 provides the flowchart for this procedure. R-CLAN and separation-index procedures complement one another and were used to measure robustness objectively.

## Results

**Fig. 3:**
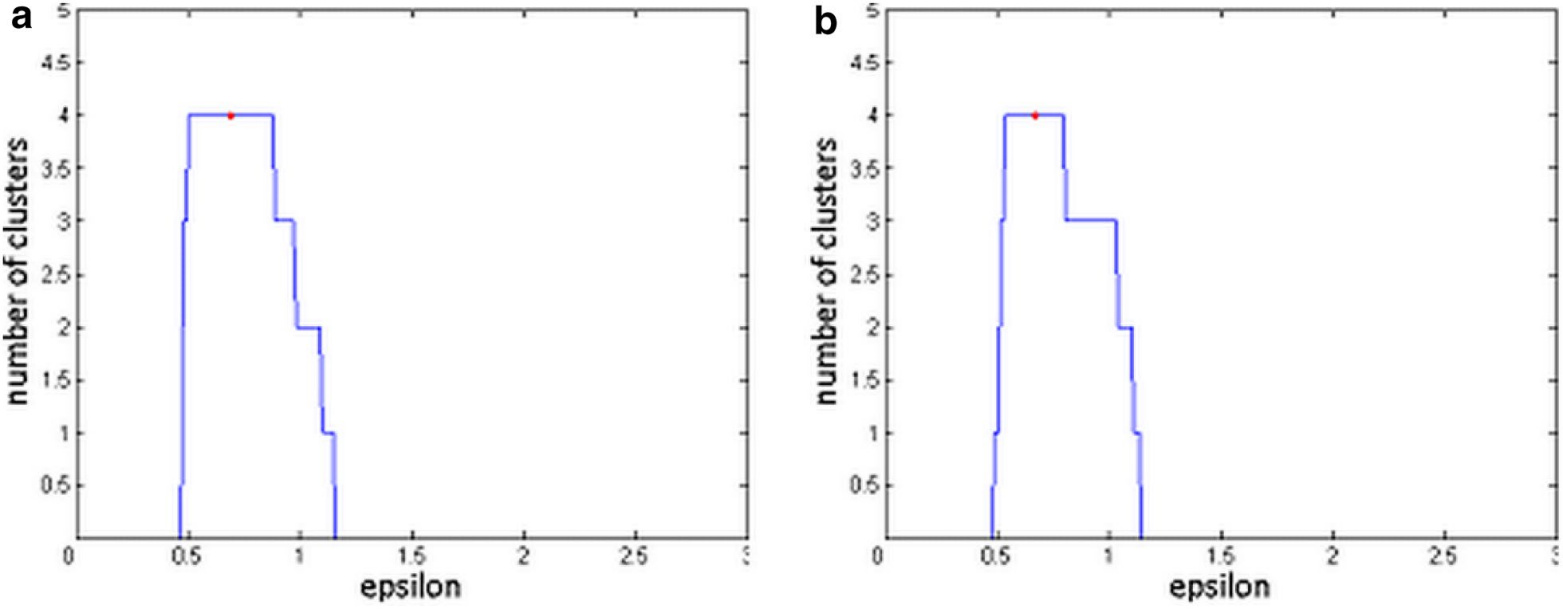
Parameter choice in DBSCAN clustering: Epsilon plot for (**a**) SFC, and (**b**) DFC. The red dot refers to the final chosen epsilon value

We first present the findings from DBSCAN and OPTICS clustering, before reporting the results from the assessment of robustness using our R-CLAN and separation index techniques. Figure 3a, b shows the epsilon plots generated using DBSCAN with SFC and DFC features, respectively. With both plots, our method of choosing *ε* resulted in DBSCAN detecting a total of four clusters, which was the expected number, given that we had four diagnostic groups.

Clustering performances are presented in Table 2. With DBSCAN clustering, the average group-wise cluster purity with SFC and DFC features were 75% and 87.88%, respectively; while OPTICS clustered with 93.18% cluster purity using SFC and 95.46% using DFC features. From this, it is clear that, (i) OPTICS performed better than DBSCAN, (ii) DFC features resulted in higher performance than SFC features, (iii) OPTICS clustering using DFC features resulted in the overall best performance, and (iv) the performance with control and AD groups were higher than that with the intermediate EMCI and LMCI groups.

**Table 2:** Success rate of clustering for each group and each feature, for both DBSCAN and OPTICS

	Success rate of clustering				Row-wise average %
	DBSCAN		OPTICS		
	SFC %	DFC %	SFC %	DFC %	
Control	80	97.14	97.14	100	*93.57*
EMCI	73.53	79.41	100	91.18	*86.03*
LMCI	64.71	82.35	82.35	91.18	*80.15*
AD	82.76	93.10	93.10	100	*92.24*
Mean	*75*	*87.88*	*93.18*	*95.46*	

Row-wise averages (last column) and column-wise averages (last row), shown in italics, provide summary statistics

The execution times for a single instance of DBSCAN and OPTICS algorithms on our computer (Intel^©^ Xeon^©^ quad-core processor, 3.5 GHz) were 0.0206 s and 0.0204 s, respectively, which is impressive. These numbers are interesting, because the developers of OPTICS (Ankerst et al. [38]) reported that OPTICS was consistently 1.6 times slower than DBSCAN. However, their observations may not be applicable to today’s computers since they assessed it in their computer 20 years ago. It should be noted that the time complexity of both algorithms is dependent on *ε* and the underlying data structure, and the general runtime complexity is the same for both: O(n*log(n)). Hence, to determine any consistent differences in execution time, the two algorithms must be repeatedly run on different datasets.

### Assessing robustness using additive noise

As noted earlier, higher cluster purity does not necessarily imply its robustness to noise or outliers. We devised a novel technique to assess the robustness of clustering (R-CLAN). A lower SNR value identified by R-CLAN indicates that the clustering method is more robust, because it would have delivered the same clustering performance even at higher noise levels. We applied this technique to clustering of both SFC and DFC features using both DBSCAN and OPTICS (see Table 3). Clearly, OPTICS clustering was more robust to noise than DBSCAN since lower SNR values are indicative of higher robustness. We obtained higher robustness with both SFC and DFC features using OPTICS, indicating that OPTICS could deliver the same level of clustering performance even at higher noise levels. OPTICS could withstand noise levels of 125–315 times (or 21–25 dB) more than what DBSCAN could. It was also observed that DFC features were more robust to noise compared to SFC features, and the best performance was delivered by OPTICS using DFC features, with an SNR of 21 dB. DFC features could withstand about 20 times (or 13 dB) more noise than SFC features using OPTICS. With these observations, we concluded that OPTICS is a more robust clustering technique than DBSCAN, and that DFC features could result in more robust clustering performance than SFC features. These findings also corroborate with clustering results presented earlier, wherein OPTICS and DFC resulted in better cluster purities than DBSCAN and SFC. Our findings attribute both superior performance and higher robustness to OPTICS clustering using DFC features.

**Table 3:** SNR values obtained as a measure of robustness

	SFC	DFC
DBSCAN	55	46
OPTICS	34	21

Lower value indicates better performance

### Separation index to evaluate robustness of reachability plot in OPTICS clustering

The reachability plot determines the quality of OPTICS clustering, and hence we devised a measure called the separation index to evaluate the robustness of the reachability plot. A higher value indicates superior robustness. Upon evaluating the measure with both SFC and DFC features (see Table 4), we found that DFC features provided a higher value of separation index, which in turn indicates that DFC, compared to SFC features, is a more robust measure for performing clustering using OPTICS. This result corroborates with the result obtained from R-CLAN regarding robustness using additive noise, wherein the DFC features resulted in higher robustness compared to the SFC features. These findings further reiterate that OPTICS is a better clustering algorithm than DBSCAN for unsupervised clustering of fMRI connectivity features obtained from subjects with cognitive impairment, and that DFC features would result in better clustering performance than SFC features.

**Table 4:** Separation index as a measure of OPTICS robustness

	Group-wise value of separation index				Mean
	Control	EMCI	LMCI	AD	
SFC	3.2914	3.6381	3.1946	3.2487	3.3432
DFC	4.4227	3.8152	3.4321	4.1152	3.9463

Higher value indicates better performance

## Discussion

In this work, density-based clustering was performed on static and dynamic functional connectivity features obtained from fMRI data of subjects with cognitive impairment. The data consisted of subjects with early and late mild cognitive impairment, Alzheimer’s disease and matched aged healthy controls. DBSCAN and OPTICS clustering techniques were applied to SFC as well as DFC features obtained from fMRI data. Since the input data came from four diagnostic categories, we expected to identify four clusters using DBSCAN and OPTICS, although the algorithms were not biased with this information a priori. Upon clustering, we found that both DBSCAN and OPTICS resulted in four clusters, obtained in a blind unsupervised manner. This shows that these techniques were able to detect inherent disease clusters from neuroimaging data without any supervision.

We found that higher cluster purities were obtained with OPTICS clustering compared to DBSCAN, and that DFC features always resulted in superior clustering performance compared to SFC features. Furthermore, the same trend was observed with the measures of robustness and separation index. OPTICS clustering combined with DFC features resulted in highest performance as well as most robustness to noise. Previous work by Jia et al. [46] reported a similar superior performance of DFC over SFC, wherein they determined that DFC features explained significantly more variance in human behavior than SFC. Another report by Jin et al. [47] found that DFC features have better ability in predicting psychiatric disorders (such as post-traumatic stress disorder) compared to SFC features. Our findings corroborate with these previous reports.

Referring to Table 2, the average clustering performance was found to be higher in control (93.57%) and AD groups (92.24%), as compared to the intermediate EMCI (86.03%) and LMCI (80.15%) groups. This indicates a rather expected trend wherein the control (completely healthy) and AD (completely ill) groups are on the extreme, resulting in less outliers, as compared to EMCI and LMCI groups that form the mid-band of the spectrum, resulting in more outliers and lower performance.

A novel technique using additive noise (called R-CLAN) was devised by us to assess the robustness of DBSCAN and OPTICS clustering. Table 3 shows that OPTICS clustering was more robust to noise than DBSCAN for both SFC and DFC feature sets, demonstrating that OPTICS would be able to withstand higher noise levels than DBSCAN. In addition, clustering with DFC features resulted in superior clustering performance as well as superior robustness to noise than clustering with SFC features. Static connectivity is popular in neuroimaging research and is extensively used, while dynamic connectivity is a newer technique that has gained traction more recently [42]. Both SFC and DFC provide characteristically distinct information. While the former provides the strength of connectivity over the entire fMRI scan, the latter provides the temporal variability of connectivity (i.e. change in connectivity over time). Recent studies have demonstrated the unique and superior properties of DFC over SFC [46, 55]. A comparison of SFC and DFC in their ability to identify hidden structures in the data is one of the novel contributions of our work, wherein DFC was found to be better and more robust than SFC. This is important, because the research community still largely prefers to not look beyond static connectivity. We hope our findings encourage researchers to incorporate dynamic connectivity analysis among their research strategies.

Traditionally, supervised classifiers are termed ‘robust’ when the testing error is close to the training error–that is, the classifier is able to perform well even on different datasets on which it is previously not trained. Our definition of robustness works in a similar manner: by adding noise to the underlying data, our form of robustness is a measure of how much the quality of data, evaluated by SNR, must change before the clustering changes the subject classes. The ‘different’ data here is not a completely new set of data, but the same data changed by noise. Besides the input parameters, feature selection and other algorithmic characteristics, an unsupervised learning algorithm would be sensitive to outliers in the data as well as underlying data quality. Since OPTICS and DBSCAN are already designed to detect outliers [37, 38], our robustness measures are a novel way of evaluating their sensitivity to data quality. According to data mining literature, this is the difference between robustness to class noise (identifying outliers [56]), and to attribute noise (errors in the feature values themselves [57]). Attribute noise tends to be more difficult to detect and eliminate than class noise [58]; hence, it is important that clustering is able to work well in the presence of high attribute noise, as it is common in fMRI data even after pre-processing and noise reduction [39]. To the best of our knowledge, little research has been done in determining the level of attribute noise in fMRI data that either clustering or a supervised classifier can withstand before losing accuracy. Using our robustness measures, results indicate that OPTICS and DFC features are the best combination of clustering method and input features, respectively, to use in the presence of attribute noise, as compared to DBSCAN and SFC features.

During our evaluation of robustness, we modeled the noise present in SFC and DFC features using additive white Gaussian noise. It is notable that modeling noise in fMRI with a white Gaussian distribution is well documented [40, 41], supported by the fact that non-biological noise comes from various independent sources [40], which tend to resemble white noise. In addition, Chen and Tyler [40] showed that the power spectrum of non-biological noise approached the flat line characteristic of a white noise spectrum, and hence could be well-approximated by white Gaussian noise; however, biological noise was non-Gaussian. Other works indicate that the Gaussian noise assumption is appropriate for data with high SNR values, but noise approaches a Rician distribution at lower SNR values [39, 59, 60]. Thus, there may be a weakness in our current model of noise since it does not consider non-white sources of noise that can corrupt features. When developing a noise reduction method on fMRI data obtained from Alzheimer’s disease patients, Garg et al. [41] used additive white Gaussian noise and Rician noise separately to model different levels of noise in artificial and real-world data [41]. Likewise, future work could compare additive Rician noise and white Gaussian noise within the R-CLAN framework. The response to clustering with various levels of different types of attribute noise could be investigated as well.

The idea of measuring noise robustness of clustering techniques is not alien to the literature. For example, in the original work that presented OPTICS, a steepness value was used to determine at which point clusters begin and end [38]. While it worked well in theory, its success was found to depend highly on the steepness value, an input parameter with no standard way of determining its appropriate value. In another method [61], similar to the peaks we used in finding the separation index, all significant local maxima in the plot were found, and significance was used to distinguish deep valleys representing well-formed clusters from shallow regions that were simply noise. The separation index is similar to these aforementioned methods, in that it is a measure of how well defined each cluster is with respect to the noise points surrounding it. However, it is a much simpler method and requires no human inputs. It could also be used in conjunction with the previous methods and other cluster extracting methods to determine how well-separated a specific cluster is from the points that form its boundaries on the reachability plot. In addition, separation index could be used as a measure of relative density of each cluster, since higher separation indices usually correspond to deeper valleys, which in turn indicates high-density regions within the dataset.

It is interesting to note that DBSCAN and OPTICS were found to be just as competitive in classifying Alzheimer’s disease patients as traditional supervised learning classifiers [62]. These two density-based clustering algorithms automatically detect outliers as part of their function, leaving us able to test for robustness to data quality–something that is difficult to do with supervised classifiers since outlier noise is more harmful to their performance [58]. In addition, unsupervised algorithms such as DBSCAN and OPTICS can be used in applications beyond just classification. They can also be used to determine hidden disease states or sub-states that are often undetected in traditional diagnostic classification, future prediction of diagnostic status of new subjects based on current clusters [63] and hypothesis generation and testing, to name a few. For example, with cerebrospinal fluid data, structural MRI and FDG-PET scans as features, an earlier study used hierarchical clustering on healthy controls to identify subgroups within these subjects that could later be susceptible to Alzheimer’s disease [64]. However, the number of clusters had to be chosen through visual assessment prior to clustering. Our results indicate that similar experiments could be performed using density-based clustering methods that require few input parameters, and no requirement to provide the number of clusters.

We used static and dynamic functional connectivity in this work. The brain networks obtained from them can also be used in a graph-theoretic framework and future studies could attempt clustering of network properties to obtain newer insights.

Supervised algorithms such as support vector machine (SVM) are popular in brain imaging. Backed by results of this study, we encourage researchers to consider density-based clustering methods in subject grouping and classification. Future work in this area could involve performing these analyses on various neurological and psychiatric disease conditions such as epilepsy, attention deficit hyperactivity disorder (ADHD), schizophrenia, autism spectrum disorder (ASD), post-traumatic stress disorder (PTSD), etc. to determine if disease classification findings are consistently good across various clinical populations.

Our main contributions are as follows: (i) we demonstrated that unsupervised learning, which is not prone to overfitting, could be used to determine inherent disease clusters that provide performances comparable to supervised learning. (ii) We demonstrated that density-based clustering methods, which require minimal input parameters, perform satisfactorily, and that the OPTICS technique is the suitable choice for fMRI connectivity data. (iii) We proposed two novel robustness assessment techniques and demonstrated that OPTICS clustering is a superior and noise-robust technique for fMRI connectivity data. (iv) For the first time in the literature, we assessed and compared the ability of static and dynamic connectivity features in identifying inherent disease clusters and found dynamic connectivity features to be superior and more robust. This is a significant finding given that the research community is still largely inclined towards the continued use of static connectivity alone.

## Conclusion

Unsupervised learning algorithms present some key theoretical advantages compared to supervised learning algorithms (e.g. a priori diagnostic labeling is not required, and they possess the potential to detect unknown data structures). Our work presented and contrasted two relatively new methods to perform unsupervised clustering on fMRI data in a relatively large clinical dataset. We briefly described and assessed the performance of two density-based clustering algorithms: DBSCAN and OPTICS. These algorithms were used to cluster three stages of cognitive impairment (EMCI, LMCI and AD) and matched healthy controls using static and dynamic functional connectivity features. DBSCAN was found to be relatively more sensitive to noise and less precise, whereas OPTICS accurately identified all four groups and was more robust to noise as measured from our proposed R-CLAN and separation index robustness measures. OPTICS clustering using DFC features was found to be more dependable than DBSCAN and SFC features. With the superiority of DFC and OPTICS, we encourage researchers to incorporate dynamic connectivity analysis among their research strategies and hope that we have motivated the community sufficiently to consider employing OPTICS clustering for subject classification and identifying hidden disease clusters.

## Supplementary information

**Additional file 1.** Supplemental information.

## Data Availability

The datasets used in this work were taken from the publicly available ADNI database, which is available for download from their website (https://www.loni.ucla.edu/ADNI).

## Abbreviations

AD: Alzheimer’s disease
ADNI: Alzheimer's disease neuroimaging initiative
DBSCAN: Density-based spatial clustering of applications with noise
DFC: Dynamic functional connectivity
EMCI: Early mild cognitive impairment
FC: Functional connectivity
fMRI: Functional magnetic resonance imaging
LMCI: Late mild cognitive impairment
MCI: Mild cognitive impairment
OPTICS: Ordering points to identify the clustering structure
R-CLAN: Recursive-clustering using additive-noise
rs-fMRI: Resting-state functional magnetic resonance imaging
SFC: Static functional connectivity
SNR: Signal-to-noise ratio
SVM: Support vector machine
