# Supplementary material for: Density-based clustering of static and dynamic functional MRI connectivity features obtained from subjects with cognitive impairment
Source: Brain Inform. 2020 Nov 26;7(1):19. doi: 10.1186/s40708-020-00120-2 (PMC7691406; doi:10.1186/s40708-020-00120-2)
Supplement: Supplementary file 1 — Additional file 1. Supplemental information. [file 40708_2020_120_MOESM1_ESM.pdf]

ADDITIONAL FILE

Density Based Clustering of Static and Dynamic Functional MRI Connectivity Features Obtained from Subjects with Cognitive Impairment

D Rangaprakash[1,2,3], Toluwanimi Odemuyiwa[4], Narayana Dutt D[5] and Gopikrishna Deshpande[6,7,8,9,10,11,12,13*], Alzheimer's Disease Neuroimaging Initiative

[1] *Athinoula A. Martinos Center for Biomedical Imaging, Massachusetts General Hospital, Charlestown, Massachusetts, USA*

[2] *Department of Radiology, Harvard Medical School, Boston, Massachusetts, USA*

[3] *Division of Health Sciences and Technology, Harvard University and Massachusetts Institute of Technology, Cambridge, Massachusetts, USA*

[4] *Division of Engineering Science, Faculty of Applied Science & Engineering, University of Toronto, Ontario, Canada*

[5] *Department of Medical Electronics, Dayananda Sagar College of Engineering, Bangalore, India*

[6] *AU MRI Research Center, Department of Electrical and Computer Engineering, Auburn University, Auburn, Alabama, USA*

[7] *Department of Psychology, Auburn University, Auburn, Alabama, USA*

[8] *Alabama Advanced Imaging Consortium, University of Alabama Birmingham, Alabama, USA*

[9] *Center for Health Ecology and Equity Research, Auburn University, Auburn, Alabama, USA*

[10] *Center for Neuroscience, Auburn University, Auburn, Alabama, USA*

[11] *School of Psychology, Capital Normal University, Beijing, China*

[12] *Key Laboratory for Learning and Cognition, Capital Normal University, Beijing, China*

[13] *Department of Psychiatry, National Institute of Mental Health and Neurosciences, Bangalore, India*

Please refer to section 2.2 of the main paper for the context of the information provided here. From the 3D+Time fMRI data, mean fMRI time series were extracted from 200 functionally homogeneous brain regions of interest (ROIs) obtained through spectral clustering (cc200 template, [1]). Table S1 provides information about these 200 ROIs.

Table S1. *ROIs of the cc200 template [1], and their MNI coordinates*

ROI no.	MNI Coordinates			ROI name (Harvard-Oxford)
	X	Y	Z	
1	-41	-83	1	Lateral Occipital Cortex
2	-51	-59	22	Angular Gyrus
3	13	-63	23	Precuneous Cortex
4	-38	-12	-3	Insular Cortex
5	-7	48	7	Paracingulate Gyrus
6	8	-34	46	Cingulate Gyrus
7	54	-46	40	Angular Gyrus
8	-41	-19	53	Precentral Gyrus
9	-20	-60	-27	Left Cerebellum
10	42	-53	-34	Cerebellar Tonsil
11	-59	-28	-4	Superior Temporal Gyrus
12	27	61	-3	Frontal Pole
13	2	14	48	Paracingulate Gyrus
14	52	-53	22	Angular Gyrus
15	13	15	-8	Subcallosal Cortex
16	-50	-64	-6	Lateral Occipital Cortex
17	45	11	31	Precentral Gyrus
18	11	-18	8	Right Thalamus
19	-11	-65	22	Precuneous Cortex
20	-34	21	1	Insular Cortex
21	-10	-36	68	Postcentral Gyrus
22	6	45	5	Cingulate Gyrus
23	-43	43	13	Frontal Pole
24	-60	-25	15	Planum Temporale
25	44	43	14	Frontal Pole
26	43	-72	-12	Lateral Occipital Cortex
27	-31	-3	-33	Parahippocampal Gyrus
28	29	-32	62	Postcentral Gyrus
29	0	-15	34	Cingulate Gyrus
30	2	-26	-37	Brain-Stem
31	29	-53	59	Superior Parietal Lobule
32	42	12	-38	Temporal Pole

33	-58	-42	25	Supramarginal Gyrus
34	-46	10	31	Middle Frontal Gyrus
35	40	-3	10	Insular Cortex
36	-41	-51	-35	Cerebellar Tonsil
37	-14	-30	0	Left Thalamus
38	41	26	37	Middle Frontal Gyrus
39	61	-40	-9	Middle Temporal Gyrus
40	1	35	23	Cingulate Gyrus
41	29	-38	-33	Cerebellar Tonsil
42	-29	52	-14	Frontal Pole
43	-40	8	-40	Temporal Pole
44	13	-93	1	Occipital Pole
45	-9	-16	9	Left Thalamus
46	1	-37	31	Cingulate Gyrus
47	-15	15	-4	Left Caudate
48	18	-32	-3	Parahippocampal Gyrus
49	61	-19	-17	Middle Temporal Gyrus
50	-29	-5	57	Precentral Gyrus
51	-1	41	-13	Frontal Medial Cortex
52	-7	-44	-40	Brain-Stem
53	44	32	-11	Frontal Orbital Cortex
54	-7	-85	24	Cuneal Cortex
55	0	18	32	Cingulate Gyrus
56	-53	-49	41	Supramarginal Gyrus
57	-29	33	-16	Frontal Orbital Cortex
58	1	-51	14	Precuneous Cortex
59	36	20	3	Insular Cortex
60	42	-18	53	Precentral Gyrus
61	-40	22	41	Middle Frontal Gyrus
62	33	-29	-17	Temporal Fusiform Cortex
63	-44	-51	-20	Inferior Temporal Gyrus
64	28	1	56	Middle Frontal Gyrus
65	12	-43	67	Postcentral Gyrus
66	61	-15	11	Planum Temporale
67	-29	3	3	Left Putamen
68	0	-19	-9	Brain-Stem
69	59	-42	10	Supramarginal Gyrus
70	-12	-69	5	Intracalcarine Cortex
71	29	26	-16	Frontal Orbital Cortex
72	-58	-13	-17	Middle Temporal Gyrus
73	-44	3	47	Precentral Gyrus
74	-45	35	-9	Frontal Orbital Cortex

75	30	57	14	Frontal Pole
76	-9	-32	45	Cingulate Gyrus
77	16	-78	-30	Pyramis
78	-42	15	-29	Temporal Pole
79	0	2	41	Cingulate Gyrus
80	-38	-69	-33	Inferior Semi-Lunar Lobule
81	13	-76	38	Precuneous Cortex
82	-44	-65	39	Lateral Occipital Cortex
83	46	9	-11	Temporal Pole
84	-14	7	13	Left Caudate
85	46	-71	15	Lateral Occipital Cortex
86	8	-42	-38	Brain-Stem
87	36	-11	-27	Temporal Fusiform Cortex
88	-59	-6	25	Postcentral Gyrus
89	7	-72	5	Intracalcarine Cortex
90	-18	-14	69	Precentral Gyrus
91	0	54	25	Superior Frontal Gyrus
92	-20	-5	-17	Left Amygdala
93	40	-41	48	Superior Parietal Lobule
94	13	1	16	Right Caudate
95	-27	14	55	Middle Frontal Gyrus
96	-30	-34	61	Postcentral Gyrus
97	-35	-80	25	Lateral Occipital Cortex
98	59	1	25	Precentral Gyrus
99	-59	-47	-9	Middle Temporal Gyrus
100	47	-51	-18	Inferior Temporal Gyrus
101	-52	-2	-29	Middle Temporal Gyrus
102	35	-85	9	Lateral Occipital Cortex
103	-29	-38	-31	Cerebellar Tonsil
104	-10	64	13	Frontal Pole
105	17	-54	2	Lingual Gyrus
106	27	36	41	Frontal Pole
107	54	-27	-1	Superior Temporal Gyrus
108	-7	-91	1	Occipital Pole
109	1	58	-8	Frontal Pole
110	30	8	-20	Temporal Pole
111	1	-22	67	Precentral Gyrus
112	-30	14	-18	Frontal Orbital Cortex
113	42	51	-6	Frontal Pole
114	-27	-72	40	Lateral Occipital Cortex
115	43	3	49	Precentral Gyrus
116	-57	-27	36	Supramarginal Gyrus

117	-57	-47	8	Middle Temporal Gyrus
118	20	-57	-26	Dentate
119	55	7	5	Central Opercular Cortex
120	-19	-79	-32	Pyramis
121	-41	-11	12	Central Opercular Cortex
122	-29	-36	-12	Parahippocampal Gyrus
123	0	-13	51	Precentral Gyrus
124	27	46	-15	Frontal Pole
125	-31	40	33	Frontal Pole
126	35	-71	-31	Tuber
127	30	20	51	Middle Frontal Gyrus
128	60	-29	25	Parietal Operculum Cortex
129	-45	1	-15	Planum Polare
130	0	-1	-10	Hypothalamus
131	-27	-91	11	Occipital Pole
132	29	-66	46	Lateral Occipital Cortex
133	-10	49	39	Frontal Pole
134	23	-11	67	Precentral Gyrus
135	14	16	6	Right Caudate
136	-15	-64	55	Lateral Occipital Cortex
137	40	-9	-5	Insular Cortex
138	21	-68	-12	Occipital Fusiform Gyrus
139	10	65	9	Frontal Pole
140	54	-1	-27	Middle Temporal Gyrus
141	-50	25	8	Inferior Frontal Gyrus
142	17	-88	21	Occipital Pole
143	52	-60	0	Middle Temporal Gyrus
144	51	30	4	Inferior Frontal Gyrus
145	-32	-19	-19	Parahippocampal Gyrus
146	-57	-9	4	Heschl's Gyrus
147	-9	-73	40	Precuneous Cortex
148	1	-3	4	Right Thalamus
149	-1	33	44	Superior Frontal Gyrus
150	-47	-70	11	Lateral Occipital Cortex
151	-45	29	25	Middle Frontal Gyrus
152	1	-55	-13	Declive
153	57	-6	-9	Superior Temporal Gyrus
154	-52	-12	39	Postcentral Gyrus
155	20	-10	-17	Right Hippocampus
156	-35	-52	49	Superior Parietal Lobule
157	53	-10	38	Postcentral Gyrus
158	-22	-90	-13	Occipital Fusiform Gyrus

159	-37	-76	-16	Occipital Fusiform Gyrus
160	-1	22	-9	Subcallosal Cortex
161	10	3	65	Supplementary Motor Cortex
162	-10	-42	-25	Brain-Stem
163	9	-62	55	Precuneous Cortex
164	51	23	20	Inferior Frontal Gyrus
165	-55	9	17	Inferior Frontal Gyrus
166	44	-63	38	Lateral Occipital Cortex
167	-27	54	22	Frontal Pole
168	32	46	27	Frontal Pole
169	-42	51	-4	Frontal Pole
170	32	-76	30	Lateral Occipital Cortex
171	-44	-36	47	Supramarginal Gyrus
172	27	-48	-14	Fusiform Cortex
173	-9	21	59	Superior Frontal Gyrus
174	-2	-53	38	Precuneous Cortex
175	30	-86	-12	Occipital Fusiform Gyrus
176	0	-61	-35	Inferior Semi-Lunar Lobule
177	-15	-73	-11	Lingual Gyrus
178	26	3	-1	Right Putamen
179	-14	-51	-2	Lingual Gyrus
180	55	-27	43	Supramarginal Gyrus
181	45	15	-24	Temporal Pole
182	-9	2	65	Supplementary Motor Cortex
183	-28	59	4	Frontal Pole
184	-48	7	2	Central Opercular Cortex
185	43	-23	15	Heschl's Gyrus
186	12	45	45	Frontal Pole
187	13	23	59	Superior Frontal Gyrus
188	-20	-51	64	Superior Parietal Lobule
189	-28	-56	-14	Fusiform Cortex
190	15	-32	-22	Parahippocampal Gyrus
191	-23	32	46	Superior Frontal Gyrus
192	-2	-75	-24	Pyramis
193	14	58	28	Frontal Pole
194	0	-29	-19	Brain-Stem
195	8	-85	-13	Lingual Gyrus
196	-47	16	-12	Temporal Pole
197	-5	-51	57	Precuneous Cortex
198	30	1	-37	Temporal Fusiform Cortex
199	-16	-28	-20	Parahippocampal Gyrus
200	-44	-29	14	Parietal Operculum Cortex

Static functional connectivity (SFC) was obtained between all pairwise ROIs using Pearson’s correlation coefficient, giving a 200×200 SFC matrix per subject. The top-100 (i.e. lowest p-values) significant features were selected for further analysis, that is, a 132×100 (subjects × features) SFC matrix was used for clustering. Similarly, a 132×100 variance of DFC matrix was obtained, which was then used in clustering. Here, we present information about the connectivity paths which feature in list of the top-100 significant SFC and DFC connectivities. Table S2 provides the top-100 SFC connections, while Table S3 provides the top-100 DFC connections.

Table S2. *The connectivity paths which correspond to the top-100 significant SFC connectivities. Please refer to Table S1 for the names and coordinates of the corresponding ROIs numbers.*

ROI-1	ROI-2	ROI-1	ROI-2	ROI-1	ROI-2	ROI-1	ROI-2
5	2	94	36	148	36	157	34
34	14	99	63	148	47	157	94
35	14	100	70	149	90	158	34
36	34	103	94	149	91	159	34
49	27	103	101	149	108	160	42
49	28	104	91	149	129	167	15
55	31	110	35	150	12	167	38
55	32	111	15	150	90	167	39
62	28	111	109	150	94	167	76
63	2	112	110	150	95	167	150
64	2	113	1	153	12	168	34
68	3	113	3	153	34	172	14
76	28	119	54	153	89	173	27
79	8	119	57	153	90	173	28
79	57	120	28	153	94	176	63
79	70	123	76	153	95	177	28
85	57	124	116	153	148	177	108
89	27	128	14	154	12	180	8
89	28	129	1	154	34	180	88
90	14	129	14	154	89	180	89
90	15	129	70	154	90	196	19
90	28	131	95	154	94	196	30
90	36	135	84	154	148	197	34
90	62	141	35	155	49	199	88
91	62	143	116	155	141	200	113

Table S3. *The connectivity paths which correspond to the top-100 significant DFC connectivities Please refer to Table S1 for the names and coordinates of the corresponding ROIs numbers.*

ROI-1	ROI-2	ROI-1	ROI-2	ROI-1	ROI-2	ROI-1	ROI-2
21	8	114	95	167	141	181	102
22	3	117	59	168	58	182	5
26	25	117	73	170	37	182	32
37	32	117	74	170	67	183	85
45	6	118	39	170	161	184	109
47	25	118	40	171	41	187	36
47	26	118	46	171	65	187	37
49	16	131	29	172	82	187	43
49	45	131	33	174	122	187	98
52	5	131	97	174	128	187	99
52	42	132	97	175	30	187	129
53	25	135	100	175	101	187	151
68	55	140	25	175	102	187	178
70	25	143	134	175	115	188	22
70	26	143	135	175	128	188	43
75	74	151	118	176	31	188	98
76	45	151	145	176	74	189	46
80	38	152	37	176	144	189	174
86	41	153	118	177	144	192	112
91	26	157	97	178	168	192	186
98	5	157	139	178	169	194	52
98	6	162	21	179	133	194	187
103	69	162	117	179	170	196	185
104	45	165	5	179	178	197	184
113	12	165	6	180	134	200	103

References:

[1] R.C. Craddock, G.A. James, P.E. Holtzheimer, X.P. Hu, H.S. Mayberg, "A whole brain fMRI atlas generated via spatially constrained spectral clustering", Human Brain Mapping 2012, 33, 1914–1928
